# Stiff substrates increase YAP-signaling-mediated matrix metalloproteinase-7 expression

**DOI:** 10.1038/oncsis.2015.24

**Published:** 2015-09-07

**Authors:** A Nukuda, C Sasaki, S Ishihara, T Mizutani, K Nakamura, T Ayabe, K Kawabata, H Haga

**Affiliations:** 1 Transdisciplinary Life Science Course, Faculty of Advanced Life Science, Hokkaido University, Sapporo, Japan; 2 Research Center for Cooperative Projects, Hokkaido University Graduate School of Medicine, Sapporo, Japan

## Abstract

Abnormally stiff substrates have been shown to trigger cancer progression. However, the detailed molecular mechanisms underlying this trigger are not clear. In this study, we cultured T84 human colorectal cancer cells on plastic dishes to create a stiff substrate or on collagen-I gel to create a soft substrate. The stiff substrate enhanced the expression of matrix metalloproteinase-7 (MMP-7), an indicator of poor prognosis. In addition, we used polyacrylamide gels (2, 67 and 126 kPa) so that the MMP-7 expression on the 126-kPa gel was higher compared with that on the 2-kPa gel. Next, we investigated whether yes-associated protein (YAP) affected the MMP-7 expression. YAP knockdown decreased MMP-7 expression. Treatment with inhibitors of epidermal growth factor receptor (EGFR) and myosin regulatory light chain (MRLC) and integrin-α2 or integrin-β1 knockdown downregulated MMP-7 expression. Finally, we demonstrated that YAP, EGFR, integrin-α2β1 and MRLC produced a positive feedback loop that enhanced MMP-7 expression. These findings suggest that stiff substrates enhanced colorectal cancer cell viability by upregulating MMP-7 expression through a positive feedback loop.

## Introduction

Recent numerous studies have shown that the stiffness of the extracellular matrix (ECM) induces phenotypes of cell malignancy, including cell proliferation,^1^ cell spread,^2, 3^ collective migration^4^ and metastasis.^5^ Indeed, malignant breast tissues are drastically stiff compared with normal mammary tissues.^3^ These findings suggest that stiffer substrates enhance malignant tumors. However, the details of the molecular mechanisms that underlie this mechanical sensing remain unclear. The presence of integrin-dependent adhesions on stiffer substrates increases the activity of RhoA.^6^ RhoA activity promotes cell contractility through phosphorylation of myosin regulatory light chain (MRLC).^7, 8^

Matrix metalloproteinases (MMPs) are a family of zinc-dependent proteinases that play a role in degrading ECM proteins. MMP-7, which is also called matrilysin or pump-1, has a broad specificity for degrading ECM component proteins, including collagen-IV, elastin, fibronectin, vitronectin, aggrecan and laminin.^9, 10^ MMP-7 expression is upregulated in many cancers, including colorectal,^11^ gastric,^12^ breast^13^ and ovarian.^14^ MMP-7 participates in several stages of multistep carcinogenesis involving cell proliferation,^15^ invasion,^16^ metastasis^17^ and angiogenesis.^18^ In addition, MMP-7 increases the resistance to Fas-mediated apoptosis in colorectal cancer.^19^ A recent study reported that MMP-7 levels were significantly increased in stage III/IV cancers relative to both adenomas and nonmetastatic diseases.^11^

Yes-associated protein (YAP) is a transcriptional factor that plays an important role in mechanotransduction along with the transcriptional co-activator with PDZ-binding motif (TAZ). YAP/TAZ conveys the mechanical signals of ECM stiffness to many intracellular signals. YAP is activated by stiffer substrates and translocated from the cytoplasm to the nucleus.^20^ The enhanced activation of YAP leads to malignant cancer phenotypes, such as metastasis,^21^ contact inhibition resistance,^22^ proliferation and epithelial-to-mesenchymal transition.^23^ Recent studies have suggested that the activity of YAP is inhibited by phosphorylation of the serine residue that occurs in a Hippo pathway-dependent or Hippo-independent process.^20, 22^ The epidermal growth factor receptor (EGFR) is a transmembrane tyrosine kinase that is overexpressed in roughly 40% of breast carcinomas. It is strongly upregulated in numerous tumor types, and its expression correlates with an adverse prognosis.^24^

The results of these studies suggest that mechanical cues are transduced intercellularly through interactions of several proteins, including integrins, YAP, MRLC and EGFR, that then enhance tumors with poor prognoses tumors. We describe a novel molecular mechanism through which stiffer substrates increase MMP-7 expression through interactions with several proteins that might be new therapeutic targets for colorectal cancer.

## Results

### Stiffer substrates enhanced MMP-7 expression in colorectal cancer

**Figure 1:**
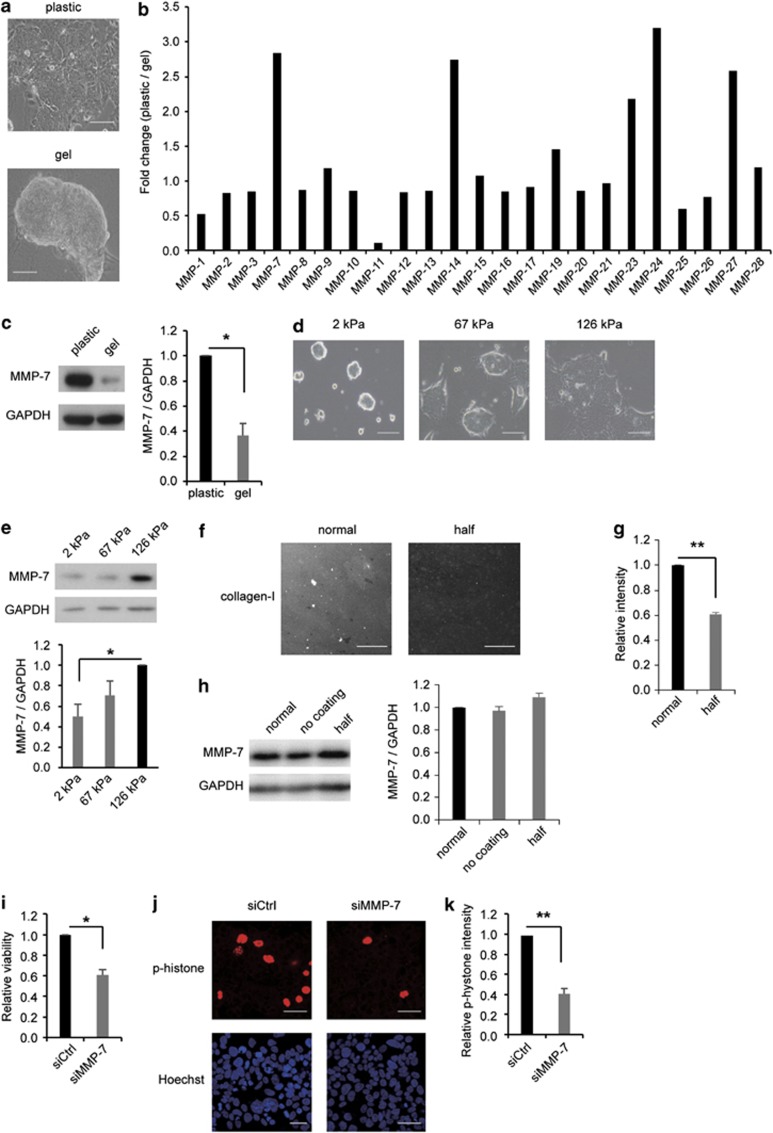
Stiffer substrates enhanced MMP-7 expression in colorectal cancer. (**a**) Cell morphology of T84 cells on a stiff substrate (plastic) or a soft substrate (gel). Scale bars, 100 μm. (**b**) The difference in the levels of MMP expression between a plastic and a gel in the microarray data. (**c**) Representative western blots (left) and quantification (right) of the MMP-7 levels on a plastic dish and collagen-I gel. (**d**) Cell morphologies of the T84 cells on 2-, 67- and 126-kPa acrylamide gels. Scale bars, 100 μm. (**e**) Representative western blots (upper) and quantification (lower) of the MMP-7 levels on 2-, 67- and 126-kPa acrylamide gels. (**f**) Immunofluorescent images of collagen-I. Scale bars, 100 μm. (**g**) Quantification of the fluorescent intensity of collagen-I on the surface of the plastic dish shown in (**f**). (**h**) Representative western blots (left) and quantification (right) of MMP-7 levels. (**i**) Effects of transfection with control (siCtrl) or MMP-7 (siMMP-7) siRNA on cell viability after 6 days. (**j**) The upper panels show phosphorylated-histone (p-histone) on a cover glass that was coated with collagen-I. The lower panels show Hoechst on a cover glass that was coated with collagen-I. Scale bars, 50 μm. (**k**) Quantification of the fluorescent intensity of p-histones after transfection with siCtrl or siMMP-7. The bars represent mean±s.e.m. *N*=at least three independent experiments. *P<0.05, **P<0.01, unpaired *t*-test.

Previous studies have suggested that abnormally stiff substrates promote the spread of the malignant phenotype of cells.^3^ We examined the morphology of T84 cells (colorectal cancer) that were remarkably changed when the cells were cultured on two different stiffness substrates (stiff or soft). When the cells were cultured on a plastic dish that was coated with collagen-I and that was used as the stiffer substrate, T84 cells spread significantly and comprised the epithelial monolayer, a malignant phenotype. Conversely, when the cells were cultured on a collagen-I gel that was used as a soft substrate, the cellular morphology exhibited the nonmalignant phenotype of a round colony and cellular aggregation (Figure 1a). Initially, in order to screen the genes that influenced substrate stiffness, we performed mRNA microarray on cells cultured on a plastic dish versus cells cultured on a gel. In the microarray data results, we focused on MMPs. Figure 1b shows the fold changes (plastic/gel ratio) in all of the MMPs that were investigated in this microarray. *MMP-7*, *MMP-14*, *MMP-23*, *MMP-24* and *MMP-27* had changes that were higher than twofold, indicating that these genes were upregulated on a plastic dish.

Next, we selected *MMP-7* as a target gene from these candidate MMPs because we found that only the *MMP-7* mRNA expression was significantly higher on plastic than that on a gel (Supplementary Figure S1a), and overexpression of MMP-7 protein is a well-known prognostic marker in colorectal cancer.^25^ We also found that the protein expression of MMP-7 on a stiff substrate was significantly higher than that on a soft substrate (Figure 1c and Supplementary Figure S1b). This result suggested that stiffer substrates enhanced MMP-7 expression in colorectal cancer. However, it was possible that a difference in the collagen-I quantity and orientation between the plastic dish and the collagen-I gel influenced the results. In order to confirm the effects of only substrate stiffness on the MMP-7 expression, we used acrylamide gels that had stiffnesses of 2, 67 or 126 kPa. We seeded T84 cells on each acrylamide gel that was coated with an equivalent amount of collagen-I. Cell morphologies on the 67- and 126-kPa acrylamide gels were similar to those observed on plastic, and the morphologies on the 2-kPa gel were similar to those on the collagen-I gel (Figure 1d). Furthermore, we found that the stiffer acrylamide gels (126 kPa) significantly enhanced MMP-7 expression compared with the expression on the 2-kPa gel (Figure 1e). Furthermore, we conducted a detailed evaluation of whether MMP-7 expression was affected by a difference in the quantity of collagen-I. We used three types of plastic for this analysis: one was coated with a normal quantity of collagen-I (normal), another was coated with half the normal quantity of collagen-I (half) and the last was not coated with ECM proteins (no coating). First, in order to quantify the difference in collagen-I on the plastic surface between the normal and half plastic surfaces, the intensity of collagen-I was evaluated using immunofluorescence analysis. The results showed that the intensity of the collagen-I signal of the half plastic was significantly downregulated compared with that of the normal plastic (Figures 1f and g). Second, we seeded the cells on the normal, half and no-coating plastics, and investigated whether MMP-7 expression was affected by the quantity of collagen-I. The results showed that MMP-7 expression was not affected by collagen-I levels (Figure 1h). These experimental results indicated that substrate stiffness regulated MMP-7 expression in colorectal cancer and that this regulation was independent of the presence of collagen-I.

Next, we assessed whether MMP-7 affected the viability of colorectal cancer cells on stiffer substrates. In order to confirm whether MMP-7 increased cell proliferation, we used MMP-7-knocked down (KD) cells that were transfected with short interfering RNA (siRNA) that targeted the MMP-7 mRNA. We compared the cell viability of the MMP-7-KD cells with negative control (NC) cells when we cultured T84 cells on a plastic dish 6 days after transfection with the siRNAs. The MMP-7-KD cells decreased cell viability compared with the NC cells, and MMP-7-KD colony diminished in size (Figure 1i and Supplementary Figure S1c). In addition, we tested histone phosphorylation in the MMP-7-KD and NC cells with immunofluorescence because histone H3 phosphorylation at Ser10 is related to mitotic chromosome condensation.^26^ The ratio of histone phosphorylation in the MMP-7-KD cells was strikingly lower than that in the NC cells (Figures 1j and k). We inferred from these results that MMP-7 promotes poor cancer prognoses by accelerating cancer cell proliferation. These findings suggested that stiffer substrates have poor prognoses because of upregulations in the expression of MMP-7 in colorectal cancer.

### YAP activity increased MMP-7 expression on stiffer substrates

**Figure 2:**
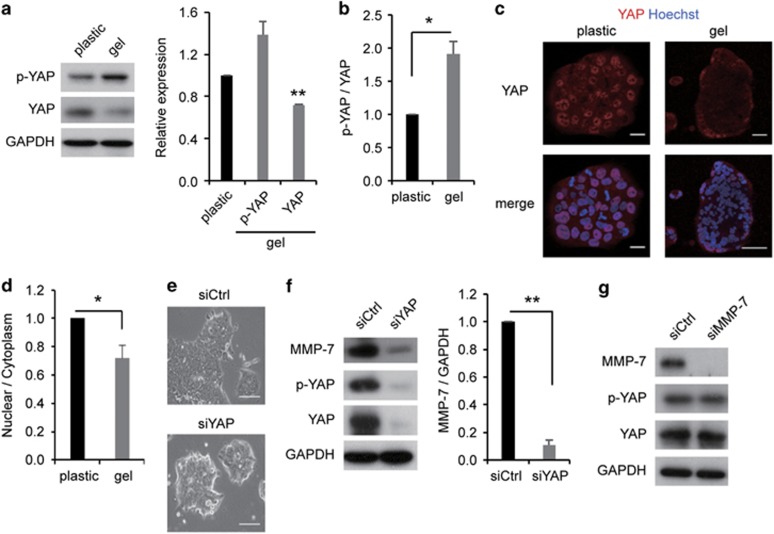
YAP activity increased MMP-7 expression on stiffer substrates. (**a**) Representative western blots (left) and quantification (right) of p-YAP and YAP on a plastic dish (plastic) or collagen-I gel (gel). (**b**) Quantification of p-YAP levels from (**a**). (**c**) The upper panels show YAP localization on a plastic or gel. The lower panels show the merged images of YAP and Hoechst on a plastic or gel. Scale bars, 50 μm. (**d**) Quantification of the fluorescent intensity of YAP in the nucleus relative to the cytoplasm from (**c**). (**e**) Cell morphology of T84 cells on a plastic after transfection with control (siCtrl) or YAP (siYAP) siRNA. (**f**) Representative western blots (left) and quantification (right) of MMP-7 levels on a plastic after transfection with siCtrl or siYAP. (**g**) Representative western blots of MMP-7, p-YAP, YAP and GAPDH on a plastic after transfection with siCtrl or siMMP-7. The bars represent mean±s.e.m. *N*=at least three independent experiments. *P<0.05, **P<0.01, unpaired *t*-test.

The results from a number of recent studies have suggested that stiffer substrates activate YAP by dephosphorylation where it accumulates in the nucleus.^20, 27^ Furthermore, activated YAP has recently been reported to regulate tumor survival.^23^ YAP is inactivated by phosphorylation, and it then accumulates in the cytoplasm.^22^ Therefore, we surveyed the phosphorylated-YAP (p-YAP) and total YAP levels in cells cultured on a plastic dish versus cells cultured on collagen-I gel. The p-YAP levels did not differ significantly, but the YAP levels on plastic were higher than those on gel (Figure 2a). In addition, we analyzed the p-YAP/YAP ratio on plastic versus gel and found that the p-YAP/YAP ratio increased on gel relative to that on plastic (Figure 2b). Furthermore, we confirmed that YAP accumulated in the nucleus on plastic compared with that on gel, and activated YAP on gel tended to accumulate in peripheral colonies compared with accumulating in the cell center (Figures 2c and d). These results indicated that YAP expression and activity were more enhanced on stiffer substrates than on soft substrates.

In order to examine whether YAP modulated the levels of MMP-7 on plastic, we transfected the cells with YAP-specific or NC siRNAs. The YAP-KD cells showed the round colony phenotype that was similar to that observed on the collagen-I gel and 2-kPa acrylamide gel (Figures 1a, d and 2e). These results suggested that YAP evoked cell spread, reflecting the malignant phenotype of T84 cells. Furthermore, the YAP-KD cells markedly suppressed the expression of MMP-7 on plastic (Figure 2f). These results indicated that YAP was a critical regulatory factor in MMP-7 expression on the stiffer substrate in colorectal cancer. To the best of our knowledge, there have been few reports on the association of YAP and MMP-7. Thus, these results are the first report of a novel mechanism of the regulation of MMP-7 expression. Next, in contrast, in order to elucidate the inverse effect of MMP-7 expression on YAP, we compared the YAP levels of the MMP-7-KD cells with the NC cells and found that MMP-7 had no effect on YAP expression and activation on stiffer substrates (Figure 2g). Together, these observations suggested that YAP is an important upstream regulatory protein of the levels of MMP-7 expression on stiffer substrates in colorectal cancer.

### MRLC regulated MMP-7 and YAP expression

**Figure 3:**
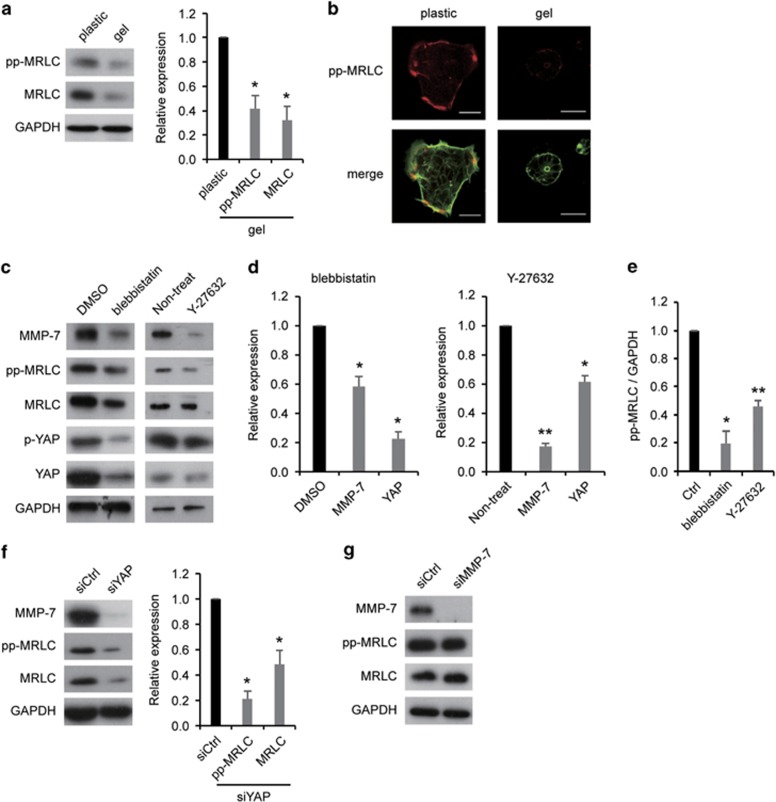
MRLC regulated MMP-7 and YAP expression. (**a**) Representative western blots (left) and quantification (right) of pp-MRLC and MRLC levels on a plastic dish (plastic) or collagen-I gel (gel). (**b**) The upper panels show pp-MRLC localization on a plastic or gel. The lower panels show merged images of pp-MRLC and F-actin on a plastic or gel. Scale bars, 50 μm. (**c**) Representative western blots of MMP-7 and YAP levels on a plastic after treatment with dimethyl sulfoxide (DMSO) or blebbistatin or after treatment with or without Y-27632. (**d**) Quantification of MMP-7 and YAP levels from (**c**). (**e**) Quantification of pp-MRLC levels from (**c**). (**f**) Representative western blots (left) and quantification (right) of MRLC and pp-MRLC levels on a plastic after transfection with control (siCtrl) or YAP (siYAP) siRNA. (**g**) Representative western blots of MMP-7, pp-MRLC, MRLC and GAPDH on a plastic dish after transfection with siCtrl or siMMP-7. The bars represent mean±s.e.m. *N*=3 independent experiments. *P<0.05, **P<0.01, unpaired *t*-test.

Next, we focused on MRLC that is known to have increased phosphorylation in lung adenocarcinoma cells on stiffer substrates^2^ and to activate YAP through the contractile actin cytoskeleton,^27^ and that is similar to the association of nonmuscle myosin II with YAP.^20, 28^ First, we validated that the phosphorylation and total levels of MRLC were increased on stiffer substrates (Figure 3a). In addition, we performed immunofluorescence of MRLC phosphorylation and F-actin that showed that MRLC phosphorylation was colocalized with lamellipodia on plastic, whereas it was slightly stained on gel (Figure 3b).

Next, we inhibited the function of myosin II and MRLC by treating them with blebbistatin, an inhibitor of nonmuscle myosin II, and Y-27632, a Rho-associated protein kinase (ROCK) inhibitor. We found that both inhibitors significantly downregulated the expression of MMP-7 and YAP on a stiff substrate (Figures 3c–e). It has not yet been reported that phosphorylated or total MRLC affects MMP-7 expression. Therefore, we report for the first time the novel mechanism of the modulation of MMP-7 expression by MRLC phosphorylation in colorectal cancer. In order to ascertain whether YAP regulated MRLC expression and/or phosphorylation, we compared the levels of MRLC and diphosphorylated-MRLC (pp-MRLC) in YAP-KD cells and NC cells. YAP-KD cells exhibited reduced total and phosphorylation levels of MRLC (Figure 3f). Furthermore, we investigated whether MMP-7 affected MRLC expression and/or phosphorylation, and MMP-7 had no relationship to the total and phosphorylation levels of MRLC (Figure 3g). Together, these results suggested that MRLC interacted with YAP and that these proteins enhanced the expression of MMP-7 on stiffer substrates in colorectal cancer.

### EGFR regulated MMP-7 expression by association with YAP and MRLC

**Figure 4:**
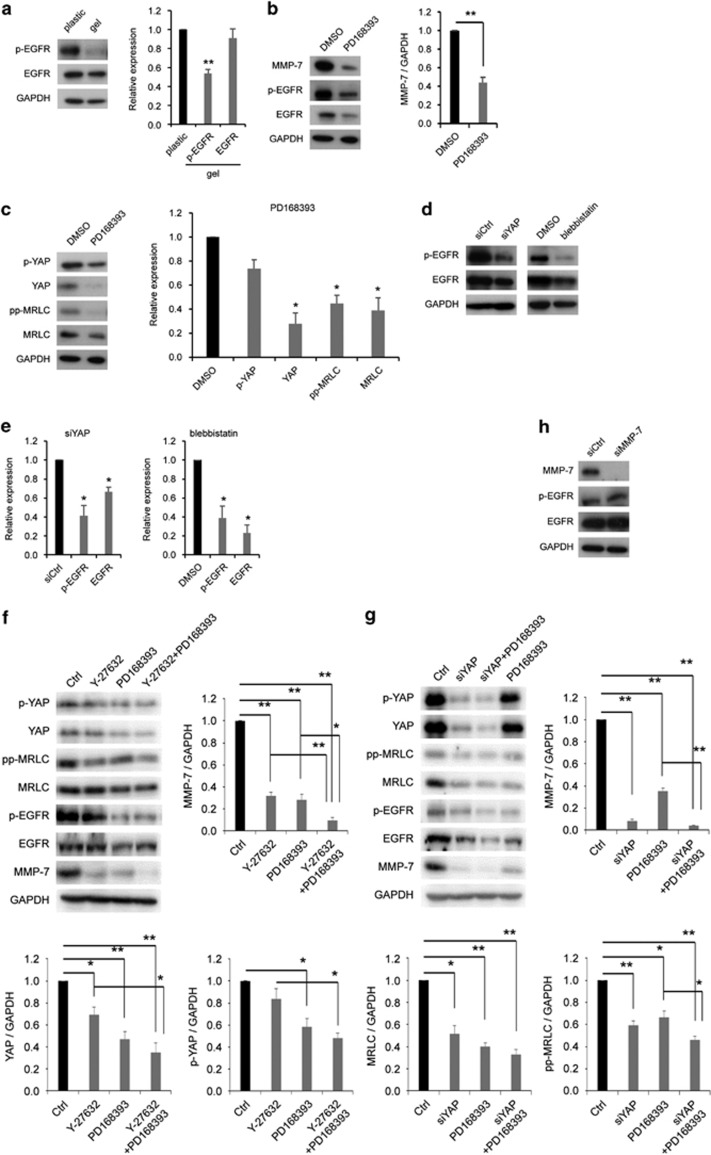
EGFR regulated MMP-7 expression by association with YAP and MRLC. (**a**) Representative western blots (left) and quantification (right) of p-EGFR on a plastic dish (plastic) or a collagen-I gel (gel). (**b**) Representative western blots (left) and quantification (right) of MMP-7 on a plastic after dimethyl sulfoxide (DMSO) or PD168393 treatment. (**c**) Representative western blots (left) and quantification (right) of p-YAP, YAP, pp-MRLC and MRLC on a plastic after DMSO or PD168393 treatment. (**d**) Representative western blots of p-EGFR, EGFR and GAPDH on a plastic after transfection with control (siCtrl) or YAP (siYAP) siRNA or after treatment with DMSO or blebbistatin. (**e**) Quantification of p-EGFR and EGFR the levels from (**c**). (**f**) Representative western blots and quantification of YAP, p-YAP and MMP-7 levels on plastic after DMSO (Ctrl), Y-27632, PD168393 or Y-27632 and PD168393 treatment. (**g**) Representative western blots and quantification of MRLC, pp-MRLC and MMP-7 levels on plastic after transfection with control (Ctrl) or YAP (siYAP) siRNA, and after DMSO or PD168393 treatment. (**h**) Representative western blots of MMP-7, p-EGFR, EGFR and GAPDH on a plastic after transfection with siCtrl or siMMP-7. The bars represent mean±s.e.m. *N*=at least three independent experiments. *P<0.05, **P<0.01, unpaired *t*-test.

Because EGFR is known to enhance MMP-7 expression in colorectal cancer,^29^ we verified whether EGFR was associated with MMP-7, YAP and MRLC on stiffer substrates. First, we found that stiffer substrates significantly activated EGFR phosphorylation (Figure 4a). Second, we blocked EGFR function by treating T84 cells with the EGFR inhibitor PD168393 on stiffer substrates. We noted that treatment with PD168393 decreased MMP-7 expression compared with treatment with dimethyl sulfoxide (Figure 4b and Supplementary Figure S2a). Previous research has revealed that YAP increases EGFR signaling by accelerating EGFR ligands.^30^ MRLC, which is required for the internalization of EGFR, induces EGFR downstream signaling, whereas EGFR enhances the phosphorylation of the Ser19 on MRLC.^31, 32^ Thus, we next examined whether the inhibition of EGFR affected YAP and MRLC expression or phosphorylation on stiffer substrates. The treatment of cells with PD168393 lowered the levels of YAP and total and phosphorylated MRLC but did not affect YAP phosphorylation levels (Figure 4c). Conversely, the cells that were transfected with siYAP or treated with blebbistatin exhibited reduced levels of total and phosphorylated EGFR (Figures 4d and e). However, the treatment of the cells with Y-27632 had the same levels of total and phosphorylation EGFR as the nontreated cells (Supplementary Figure S2b). These results indicated that nonmuscle myosin II upregulated EGFR expression, and this was independent of ROCK-mediated MRLC signaling.

Furthermore, we investigated whether EGFR directly regulates YAP expression without the involvement of ROCK and MRLC. We treated cells with only one inhibitor, Y-27632 or PD168393, or with both types of inhibitors simultaneously. The cells treated with both inhibitors exhibited decreased YAP expression compared with those treated with only Y-27632, and showed downregulated MMP-7 expression compared with cells treated with only Y-27632 or PD168393 (Figure 4f). This result suggested that EGFR directly regulates YAP and MMP-7 expression without the involvement of ROCK and MRLC, and that MRLC directly regulates MMP-7 expression without the involvement of EGFR. Furthermore, these results showed that ROCK and MRLC directly regulate MMP-7 expression without the involvement of YAP, because regardless of the difference in YAP expression between the cells treated with PD168393 and those treated with both inhibitors, MMP-7 expression in the cells treated with both inhibitors was downregulated compared with those treated with PD168393 alone. In addition, we examined whether YAP directly regulated pp-MRLC and MRLC expression without the involvement of EGFR. After the cells were transfected with siYAP and treated with PD168393, they exhibited reduced pp-MRLC and MMP-7 expression compared with cells treated with PD168393 only (Figure 4g). This result showed that YAP directly regulates pp-MRLC and MMP-7 expression without the involvement of EGFR. We finally tested whether MMP-7 regulated EGFR and/or EGFR phosphorylation with siMMP-7. The levels of EGFR expression and phosphorylation did not significantly change in the MMP-7-KD cells (Figure 4h). Together, these results suggested that EGFR, YAP, myosin II and MRLC increased MMP-7 expression by forming a positive feedback loop.

### Integrin-β1 and integrin-α2 upregulated MMP-7 expression through YAP, MRLC and EGFR

**Figure 5:**
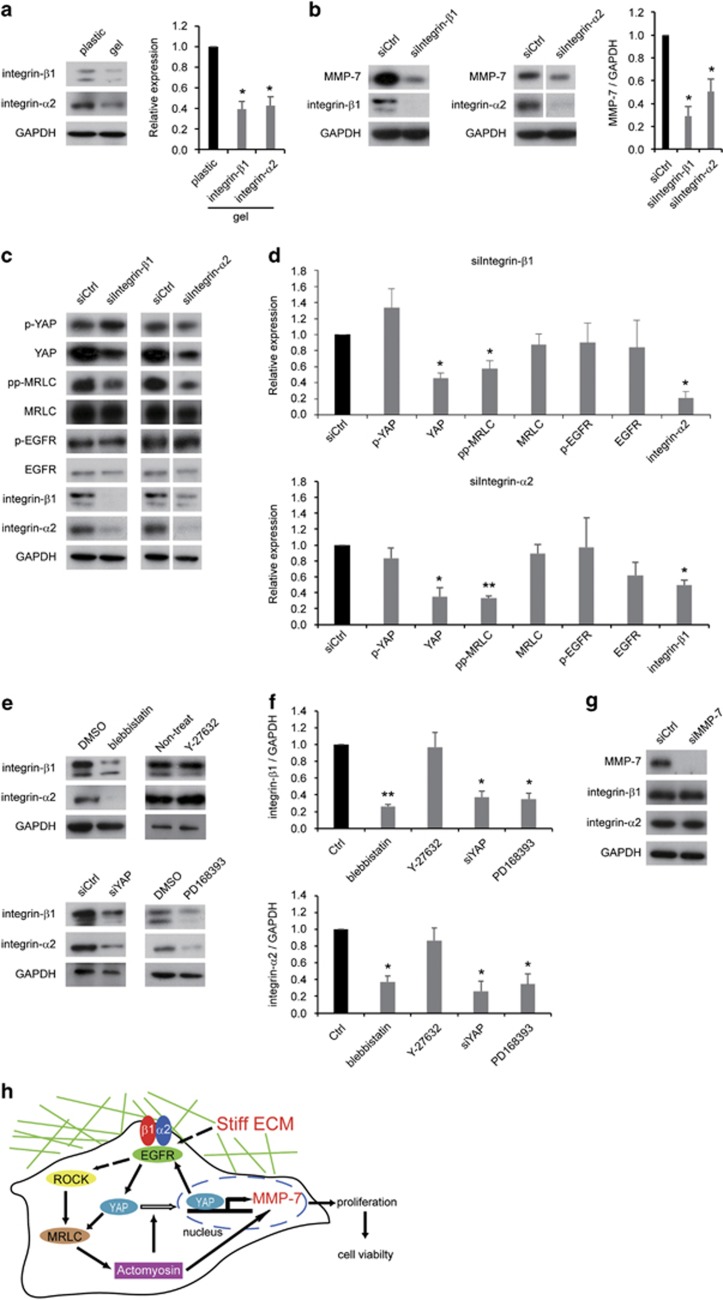
Integrin-β1 and integrin-α2 upregulated MMP-7 expression through YAP, MRLC and EGFR. (**a**) Representative western blots (left) and quantification (right) of integrin-β1 and integrin-α2 levels on a plastic dish (plastic) or a collagen-I gel (gel). (**b**) Representative western blots (left) and quantification (right) of MMP-7 levels on a plastic after transfection with control (siCtrl), integrin-β1 (siIntegrin-β1) or integrin-α2 (siIntegrin-α2) siRNA. (**c**) Representative western blots of p-YAP, YAP, pp-MRLC, MRLC, p-EGFR, EGFR, integrin-β1, integrin-α2 and GAPDH on a plastic after transfection with siCtrl, siIntegrin-β1 or siIntegrin-α2. (**d**) Quantification of p-YAP, YAP, pp-MRLC, MRLC, p-EGFR, EGFR, integrin-β1 and integrin-α2 levels from (**c**). (**e**) Representative western blots of integrin-β1, integrin-α2 and GAPDH on a plastic after transfection with siCtrl, siIntegrin-β1 or siIntegrin-α2, after treatment with dimethyl sulfoxide (DMSO), PD168393 or Y-27632 or without treatment (Non-treat) with Y-27632. (**f**) Quantification of integrin-β1 and integrin-α2 levels from (**e**). (**g**) Representative western blots of MMP-7, integrin-β1, integrin-α2 and GAPDH on a plastic after transfection with siCtrl or siMMP-7. (**h**) Stiffer substrates upregulated the levels of integrin-β1, integrin-α2 and EGFR phosphorylation and subsequently increased actomyosin contractility that was regulated by MRLC phosphorylation, resulting in YAP activation. As a result, stiffer substrates enhanced MMP7 expression, therefore generating a positive feedback loop. MMP7 accelerates cancer cell viability by enhancing cell proliferation. The bars represent mean±s.e.m. *N*=at least three independent experiments. *P<0.05, **P<0.01, unpaired *t*-test.

The association of integrin-β1 with EGFR is well known.^33^ Integrin-α2β1 associates with EGFR, and these protein interactions increase EGFR phosphorylation.^34^ Thus, we next investigated whether integrin-β1 and integrin-α2 controlled MMP-7 expression on stiffer substrates in colorectal cancer. First, we validated whether the levels of integrin-β1 and integrin-α2 were influenced by substrate stiffness, and stiffer substrates enhanced the levels of integrin-β1 and integrin-α2 (Figure 5a). Second, in order to ascertain the influence of integrin-β1 and integrin-α2 on MMP-7 expression, we downregulated the levels of integrin-β1 or integrin-α2 with specific siRNAs for each (Supplementary Figure S3a). As a result, the morphologies of the integrin-β1-KD or integrin-α2-KD cells showed round colony that were similar, especially the integrin-β1-KD colony, to the YAP-KD cell colonies (Supplementary Figure S3b). In addition, the integrin-β1-KD or integrin-α2-KD cells significantly suppressed MMP-7 expression on a stiffer substrate (Figure 5b). Because integrin-β1 and cadherins are involved in upregulating MMP-7 expression,^35^ our results were consistent with regard to the relationship of integrin-β1 and MMP-7. However, to the best of our knowledge, there have been few reports about the association of integrin-α2 with MMP-7. Therefore, we revealed for the first time that integrin-α2 increased MMP-7 expression in colorectal cancer. Activated integrin-β1 enhanced the levels of YAP/TAZ and promoted their translocation to the nucleus.^28^ Integrin-β1 has been implicated as an important mediator of ECM-induced MRLC phosphorylation.^36^ Next, in order to investigate whether integrin-β1 or integrin-α2 regulated the expression of YAP, MRLC, EGFR, integrin-β1 and integrin-α2, we examined the levels of these proteins in integrin-β1-KD or integrin-α2-KD cells. These results clarified that the integrin-β1-KD cells exhibited decreased YAP, MRLC phosphorylation and integrin-α2 expression, but the levels of YAP phosphorylation, MRLC, EGFR and EGFR phosphorylation were not changed (Figures 5c and d). The integrin-α2-KD cells exhibited downregulated levels of YAP, MRLC phosphorylation and integrin-β1, but the levels of YAP phosphorylation, MRLC, EGFR and EGFR phosphorylation were not changed (Figures 5c and d).

Conversely, we examined whether YAP, MRLC or EGFR regulated the expression of integrin-β1 or integrin-α2. The cells that were treated with blebbistatin or PD168393 and the YAP-KD cells exhibited a suppressed expression of integrin-β1 and integrin-α2, whereas the cells that were treated with Y-27632 did not exhibit changes in the levels of integrin-β1 and integrin-α2 (Figures 5e and f). Finally, we confirmed the influence of MMP-7 expression on the levels of integrin-β1 and integrin-α2 with MMP-7-specific siRNA. MMP-7-KD cells on plastic did not exhibit altered integrin-β1 or integrin-α2 expression (Figure 5g). Together, these results suggested that integrin-β1 and integrin-α2 upregulated MMP-7 expression through YAP, MRLC and EGFR by generating a positive feedback loop on stiffer substrates in colorectal cancer.

Our study demonstrated that mechanical signal transduction promoted cancer cell viability by increasing MMP-7 expression (Figure 5h). Integrins play a well-known crucial role in mechanosensing. Integrins can be responsive to an external force by changing their conformation at the plasma membrane, and can therefore function as primary mechanosensors. Mechanical stretching stimulates the conformational activation of integrin-αvβ3 and increases the binding of integrins to ECM proteins.^37^ In contrast, the phosphorylation of EGFR was immediately induced in response to attachment to the plastic. In addition, EGFR phosphorylation is mediated by integrin-β1, and these proteins form a complex.^38^ These studies suggest that integrins and EGFR are key proteins in mechanosensing, and they likely played a role as a mechanosensory complex in the present study. Therefore, stiffer substrates increased the levels of integrin-β1, integrin-α2 and activated EGFR. Furthermore, these proteins enhanced the levels of MRLC phosphorylation that subsequently activated actomyosin. This resulted in an increase in the levels of total and dephosphorylated YAP, and this YAP activation enhanced MMP-7 expression and upregulated the levels of integrin-β1, integrin-α2 and EGFR. Thus, these proteins generated a positive feedback loop that upregulated the levels of MMP-7, resulting in the promotion of cancer cell proliferation and accelerated the viability of colorectal cancer cells on stiffer substrates.

## Discussion

The extracellular environment surrounding cancer cells plays an important role in cancer development, invasion and metastasis. Indeed, it has been reported that tumor tissues were stiffer than normal mammary tissues in transgenic mice.^3^ Such an increase in substrate stiffness induced the disturbance of cytoskeletal tension and mechanotransduction that can trigger tumorigenesis and metastasis.^39^ A stiff substrate increases cytoskeletal tension by activating the Rho–ROCK pathway that induces the formation and stabilization of focal adhesion and activates focal adhesion kinase-mediated integrin signaling. In addition, the Rho–ROCK pathway also promotes EGFR–Ras–extracellular-signal-regulated kinase pathway-mediated tumor cell proliferation, and activated EGFR promotes cancer cells to resist apoptosis by activating phosphatidylinositol 3-kinase–Akt pathways. Metastatic cells have specific features that allow them to break down the basement membrane, invade the surrounding tissue, penetrate blood vessels and exit the blood vessels, leading to tumorigenesis of a new tissue. A recent study showed that matrix remodeling depending on integrin and Rho-mediated MRLC activity was required for cancer invasion in co-cultures of carcinoma cells and fibloblasts.^40^ Furthermore, adhesion to the endothelial lining of blood vessels and subsequent extravasation is required for the metastasis of tumor cells. This phenomenon is regulated by the hydrodynamic shear rate in leukocyte–tumor cells under flow conditions.^41^ Furthermore, it was recently reported that a stiff ECM promotes epithelial–mesenchymal transition and tumor metastasis by induction of TWIST1 nuclear localization.^42^ These studies suggest that a stiff substrate promotes cancer progression by affecting the multistep processes of tumorigenesis and metastasis in various cancer cell types. We demonstrated that a stiff substrate increased the expression of MMP-7, including degradation of the basement membrane, that would likely aid in the development of improved cancer therapy.

The association of integrin-β1 with EGFR and its phosphorylation is well known; however, neither the expression of p-EGFR nor EGFR was changed in integrin-KD cells. One potential reason for this result is that other integrins may possibly assist with the phosphorylation of EGFR instead of integrin-β1 in the absence of integrin-β1. Indeed, it has been reported that integrin-αvβ5, integrin-αvβ3 and integrin-α5β1 are associated with the activation of EGFR.^33, 43, 44^ When the cells were transfected with siIntegrin-β1, the depletion of integrin-β1 occurred more rapidly than the downregulation of YAP and pp-MRLC. Therefore, other integrins are likely rapidly initiated to rescue the phosphorylation of EGFR in integrin-β1-KD cells. Therefore, the phosphorylation of EGFR was recovered to baseline levels by the action of other integrins in integrin-β1-KD cells, although the other downregulated proteins were not fully recovered in these cells.

We demonstrated a novel regulatory molecular mechanism of the MMP-7 expression by YAP in colorectal cancer. However, the direct interaction between MMP-7 and YAP still remains unclear. It has been reported that the MMP-7 promoter has the consensus sequences of TATA, the activator protein-1 family, including c-Jun and c-Fos, and polyoma enhancer A binding protein-3.^45, 46^ Indeed, c-Fos and c-Jun activate the activity of the MMP-7 promoter by binding to the MMP-7 promoter, thus increasing MMP-7 expression.^46, 47^ In addition, YAP and FOS cooperatively regulate downstream genes, such as *VIM* and *Slug*, by binding to the same promoter regions.^23^ These findings indicate that YAP and MMP-7 directly interact by the binding of YAP to the MMP-7 promoter that significantly enhances MMP-7 expression. In addition, FOS and JUN probably acted as co-activators with YAP in our experimental system.

In summary, we demonstrated that stiffer substrates enhanced MMP-7 expression by generating a positive feedback loop involving EGFR, integrin-β1, integrin-α2, MRLC and YAP on stiffer substrates in colorectal cancer. In addition, we suggest that MMP-7 induces poor prognoses in colorectal cancer by promoting cell viability depending on the cell proliferation. Some previous studies have revealed that EGFR and integrin-β1 regulate MMP-7 expression; however, to the best of our knowledge, it has not been previously reported that integrin-α2, MRLC and YAP regulate MMP-7 expression in cancers. In this study, we demonstrated a novel mechanism underlying the regulation of MMP-7 expression according to substrate stiffness signaling through the association of these proteins. These findings suggest new therapeutic targets in colorectal cancer.

## Materials and methods

### Cell culture

T84 cells from the human colorectal carcinoma cell line (#CCL-248), purchased from the ATCC (Manassas, VA, USA), were cultured in a Dulbecco's modified Eagle's medium (Sigma-Aldrich Co. LLC, St Louis, MO, USA)/F-12 (Sigma-Aldrich Co. LLC) mixture that was supplemented with 10% fetal bovine serum (Biowest SAS, Nuaillé, France), MEM non-essential amino acid solution (Sigma-Aldrich Co. LLC), L-glutamine (Sigma-Aldrich Co. LLC) and 1% antibiotic/antimycotic solution (Sigma-Aldrich Co. LLC). The cells were cultured in a humidified incubator at 37 °C with 5% CO_2_.

### Materials

YAP antibody (Cell Signaling Technology, Inc., Danvers, MA, USA) and Phospho-Myosin Light Chain 2 (Thr18/Ser19) antibody (Cell Signaling Technology, Inc.) were used for immunofluorescence and western blotting. Western blotting antibodies that were specific for CD29 (integrin-β1) and CD49b (integrin-α2) were purchased from BD Biosciences (San Jose, CA, USA). Antibodies that were specific for Phospho-YAP (Ser127), EGFR and myosin light chain 2 were purchased from Cell Signaling Technology, Inc. Antibodies for MMP-7 (Daiichi Fine Chemical Co., Ltd, Toyama, Japan), EGFR (Phospho-Tyr1092; Signalway Antibody LLC, College Park, MD, USA), glyceraldehyde 3-phosphate dehydrogenase (GAPDH; Life Technologies Corporation, Grand Island, NY, USA), horseradish peroxidase anti-mouse IgG (Bio-Rad Laboratories, Inc., Hercules, CA, USA) and horseradish peroxidase anti-rabbit IgG (Cell Signaling Technology, Inc.) were used for western blotting. Alexa Fluor 488, 546 and 594 Goat Anti-Rabbit IgGs were purchased from Life Technologies Corporation. The phospho-Histone H3 (Ser10) Rabbit mAb (Cell Signaling Technology, Inc.) and Rabbit anti-Pig Collagen I and III (Cell Sciences, Inc., Canton, MA, USA) were used for immunofluorescence. MFP 488-phalloidin (MoBiTec GmbH, Göettingen, Germany) or Alexa Fluor-488 Phalloidin (Life Technologies Corporation) was used to stain F-actin, and Hoechst33342 (Sigma-Aldrich Co. LLC) was used for nuclear staining.

### Inhibitor treatment

T84 cells were seeded in a 35-mm plastic dish on day 0. On day 1, 10 μM of Y-27632 (Sigma-Aldrich Co. LLC), 25 μM of blebbistatin (Enzo Life Sciences, Inc., Farmingdale, NY, USA), 1 μM of PD168393 (EMD Millipore, Billerica, MA, USA) or dimethyl sulfoxide (Wako Pure Chemical Industries, Ltd, Osaka, Japan) was added. On day 4, the cell lysate was extracted for western blotting.

### Culture substrates

We also used plastic dishes that were coated with collagen-I (Nitta Gelatin Inc., Osaka, Japan) as stiff substrates. For soft substrates, we used 1.6 mg/ml of collagen-I gels. Polyacrylamide gels were made as described previously.^2^ Polyacrylamide gels were prepared using the following reagents (2.0 kPa: 0.01% *N*,*N*′-methylenebisacrylamide (BIS), 7.5% acrylamide and 240 mM *N*-acryloyl-6-aminocaproic acid (ACA); 67 kPa: 0.32% BIS, 7.5% acrylamide and 90 mM ACA; 126 kPa: 0.64% BIS, 7.5% acrylamide and 70 mM ACA).

### Western blotting

Cells were fixed in 10% cold trichloroacetic acid for 5 min on ice. Cells were washed 3 times with cold phosphate-buffered saline for 3 min on ice, and the cells were then lysed in SDS buffer (0.25 M Tris-HCl, 5% dithiothreitol, 2.3% SDS, 10% glycerol and 0.01% bromophenol blue, pH 6.8). The cell lysates were treated with ultrasonic fragmentation and heated at 95 °C for 5 min. Western blotting was performed as previously reported.^48^

### Immunofluorescence staining

Immunofluorescence staining of cells and image capture were performed as previously reported,^2^ but with incubation of primary antibodies overnight at room temperature, and incubation of secondary antibodies and Phalloidin with or without Hoechst33342 for 1 h at 37 °C. In the case of collagen-I staining, the coated collagen-I was fixed with 4% paraformaldehyde for 10 min at room temperature and then washed 3 times with phosphate-buffered saline. Primary and secondary antibodies in phosphate-buffered saline were incubated overnight at 4 °C or for 1 h at 37 °C. In order to quantify YAP localization, YAP intensity was calculated as a nuclear/cytoplasm ratio with ImageJ software (http://imagej.nih.gov/ij/).

### Small interfering RNA

Cells were transfected with the following siRNA or random RNA with Lipofectamine RNAiMAX Reagent (Life Technologies Corporation). Target sequences are listed in Supplementary Table S1.

### Viability assay

Cells were seeded at a density of 5 × 10^5^ on a plastic dish and transfected with siRNA against MMP-7 or random RNA (day 0). In order to prolong MMP-7 knockdown, cells were trypsinized after 3 days and transfected with siMMP-7 or random RNA (day 3). After 3 days, the cells were trypsinized, and the numbers of cells were counted 10 times with a counting chamber (day 6). The average of the 10 counts was defined (*N*=1).

### Quantitative PCR

RNA extraction, reverse transcription reaction and quantitative PCR were performed as previously reported.^48^ Primer sequences are listed in Supplementary Table S2.

### Statistical analysis

The data are shown as mean±s.e.m. Statistical analyses were performed with Welch's *t*-tests. *P-*values of <0.05 were considered statistically significant.
