# Supplementary material for: Stiff substrates increase YAP-signaling-mediated matrix metalloproteinase-7 expression
Source: Oncogenesis. 2015 Sep 7;4(9):e165–. doi: 10.1038/oncsis.2015.24 (PMC4767936; doi:10.1038/oncsis.2015.24)
Supplement: Supplementary Figure Legends [file oncsis201524x1.doc]

**Supplementary Figure legends**

**Supplementary Figure 1. Stiffer substrates enhanced matrix metalloproteinase-7 (MMP-7) expression in colorectal cancer.** (**a**) Quantification of *MMP-7, -14, -23, -24*, and *-27* mRNA expression on a plastic dish (plastic) or collagen-I gel (gel), as detected by real-time PCR. The expression of *MMP-27* on a plastic dish was not detected (ND). The bars represent mean ± SEM; n = 3 independent experiments. **P* < 0.05, unpaired *t*-test. (**b**) Representative western blots of MMP-7 and glyceraldehyde 3-phosphate dehydrogenase (GAPDH) on a plastic or gel. (**c**) Cell morphology of T84 cells on a plastic dish after transfection with control (siCtrl) or MMP-7 (siMMP-7) small interfering RNA (siRNA). Scale bars, 100 μm.

**Supplementary Figure 2. Epidermal growth factor receptor (EGFR) regulated MMP-7 expression by association with YAP and MRLC.** (**a**) Quantification of phosphorylated-EGFR (p-EGFR) levels from Figure 4b. (**b**) Representative western blots (left) and quantification (right) of p-EGFR and EGFR on a plastic dish treated with or without (Non-treat) Y-27632. The bars represent mean ± SEM. *n* = 3 independent experiments. **P* < 0.05, unpaired *t*-test.

**Supplementary Figure 3. Integrin-β1 and integrin-α2 upregulated MMP-7 expression through YAP, MRLC, and EGFR.** (**a**) Quantification of integrin-β1 and integrin-α2 levels from Figure 5b. (**b**) The cell morphology of T84 cells on a plastic dish after transfection with control (siCtrl), integrin-β1 (siIntegrin-β1), or integrin-α2 (siIntegrin-α2) siRNA. The bars represent mean ± SEM. *n* = 3 independent experiments. ***P* < 0.01, unpaired *t*-test.
