# Supplementary figures and images for: Stiff substrates increase YAP-signaling-mediated matrix metalloproteinase-7 expression
Source: Oncogenesis. 2015 Sep 7;4(9):e165–. doi: 10.1038/oncsis.2015.24 (PMC4767936; doi:10.1038/oncsis.2015.24)

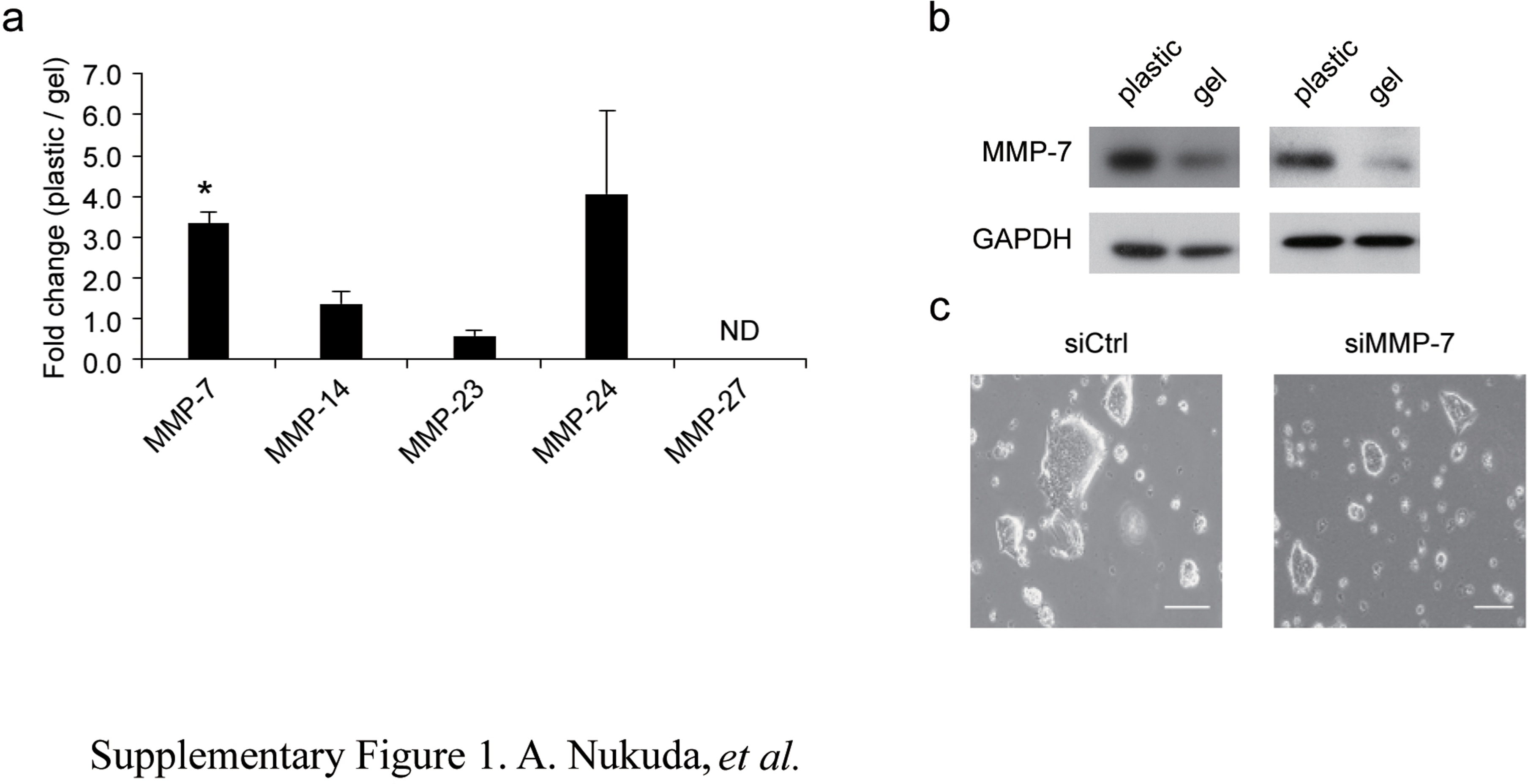

Supplement: Supplementary Figure 1 [file oncsis201524x2.tif]

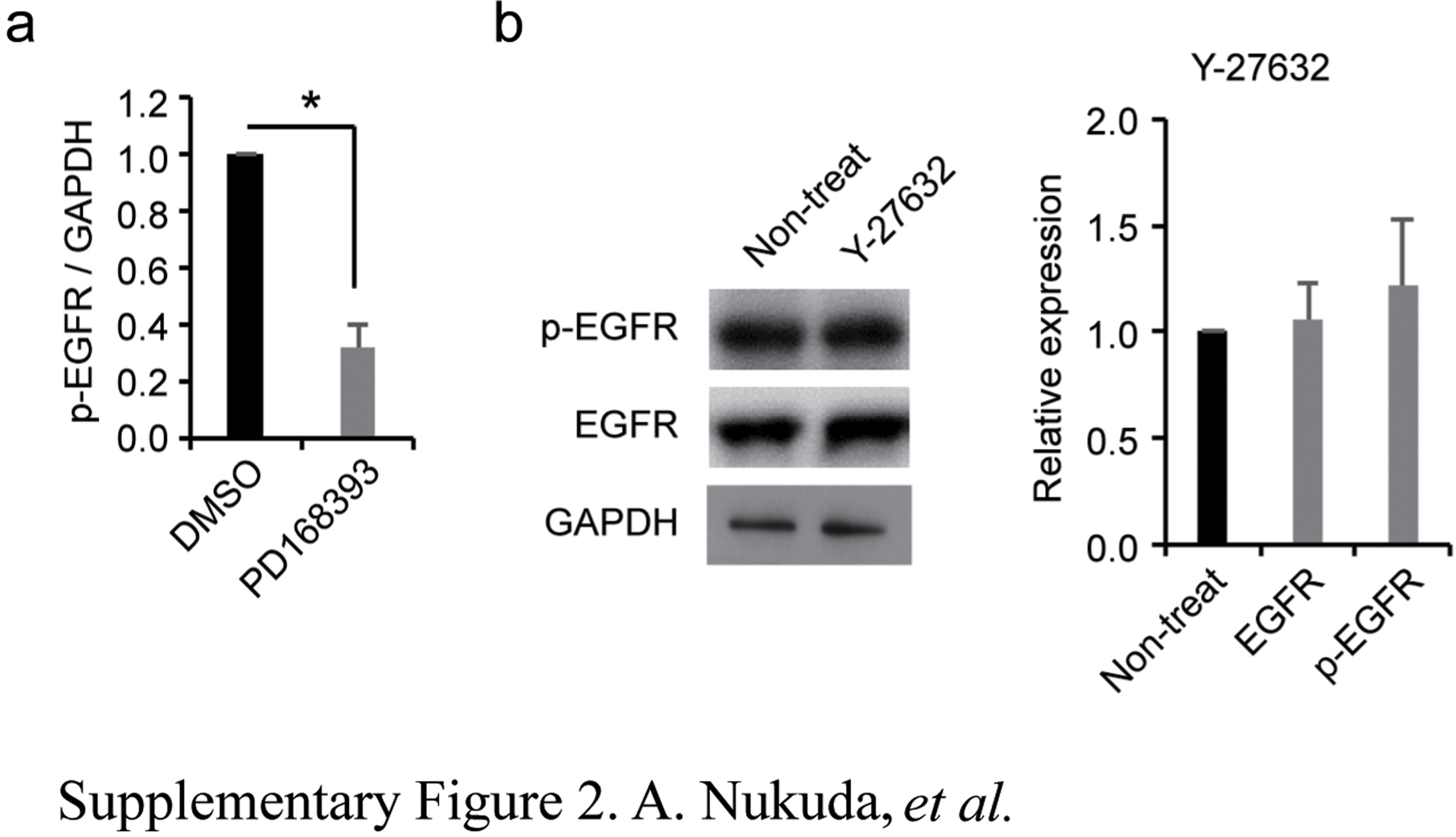

Supplement: Supplementary Figure 2 [file oncsis201524x3.tif]

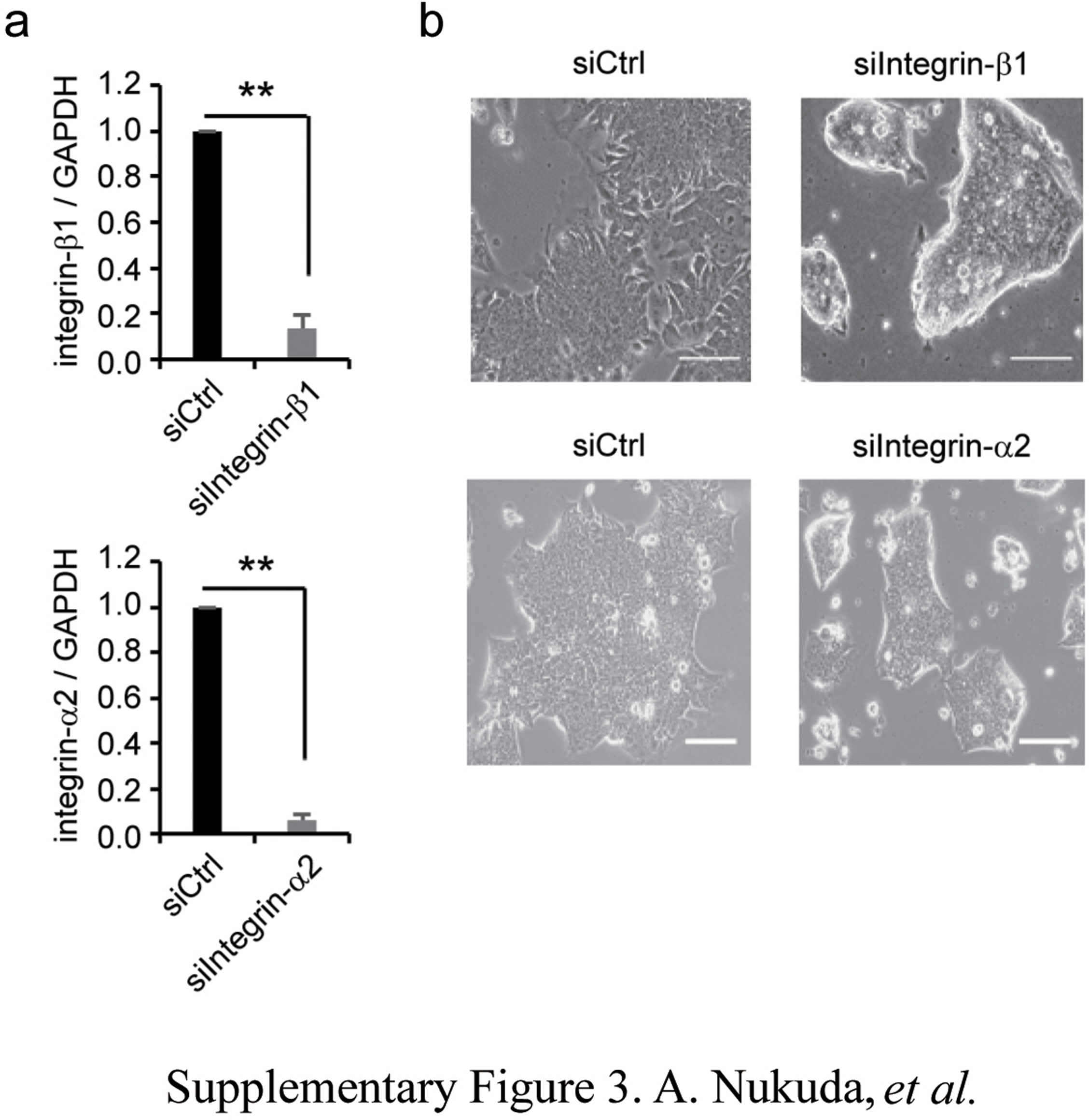

Supplement: Supplementary Figure 3 [file oncsis201524x4.tif]
